# Recent Advances in Plant NLR Structure, Function, Localization, and Signaling

**DOI:** 10.3389/fimmu.2013.00348

**Published:** 2013-10-21

**Authors:** Dong Qi, Roger W. Innes

**Affiliations:** ^1^Department of Biology, Indiana University, Bloomington, IN, USA

**Keywords:** plant innate immunity, leucine-rich repeats, disease resistance, hypersensitive response, *Pseudomonas syringae*, pathogen effectors

## Abstract

Nucleotide-binding domain leucine-rich repeat (NLR) proteins play a central role in the innate immune systems of plants and vertebrates. In plants, NLR proteins function as intracellular receptors that detect pathogen effector proteins directly, or indirectly by recognizing effector-induced modifications to other host proteins. NLR activation triggers a suite of defense responses associated with programed cell death (PCD). The molecular mechanisms underlying NLR activation, and how activation is translated into defense responses, have been particularly challenging to elucidate in plants. Recent reports, however, are beginning to shed some light. It is becoming clear that plant NLR proteins are targeted to diverse sub-cellular locations, likely depending on the locations where the effectors are detected. These reports also indicate that some NLRs re-localize following effector detection, while others do not, and such relocalization may reflect differences in signaling pathways. There have also been recent advances in understanding the structure of plant NLR proteins, with crystal structures now available for the N-terminal domains of two well-studied NLRs, a coiled-coil (CC) domain and a Toll-interleukin Receptor (TIR). Significant improvements in molecular modeling have enabled more informed structure-function studies, illuminating roles of intra- and inter-molecular interactions in NLR activation regulation. Several independent studies also suggest that intracellular trafficking is involved in NLR-mediated resistance. Lastly, progress is being made on identifying transcriptional regulatory complexes activated by NLRs. Current models for how plant NLR proteins are activated and how they induce defenses are discussed, with an emphasis on what remains to be determined.

## Introduction

Plants do not have an adaptive immune system like that found in vertebrate animals. Instead, plants depend solely on an innate immune system that bears intriguing similarities to animal innate immune systems, but is likely independently evolved [see review by Jacob et al. (1)]. Plant innate immunity is a two-tier resistance system (2). The first tier consists of plasma-membrane (PM) localized pattern recognition receptors (PRRs) that mediate detection of conserved microbial molecules referred to as pathogen associated molecular patterns (PAMPs). This type of resistance is known as PAMP triggered immunity (PTI). Most plant PRRs are transmembrane receptor kinases, with the majority containing extracellular leucine-rich repeats (LRR), thus have functional and structural similarity to the Toll-like Receptors of animals. The second tier system consists of intracellular receptors that detect the presence of pathogen proteins inside the host cell. Pathogen proteins that get inside host cells are commonly referred to as effector proteins, thus this second tier is usually referred to as effector triggered immunity (ETI).

Effector triggered immunity is mostly mediated by nucleotide-binding leucine-rich repeat (NLR) proteins. Plant NLR proteins usually contain a C-terminal LRR domain and a central NB-ARC domain (nucleotide-binding adaptor shared by Apaf-1, Resistance proteins, and CED-4) (3).The NB-ARC proteins form a subclass in the STAND super family (signal transduction ATPases with numerous domains) and function as molecular switches regulating many processes, including immunity and apoptosis (4, 5). Plant NLRs are roughly divided into two groups, depending on their N-terminal structures, CNL (CC-NB-LRR) with an N-terminal coiled-coil domain and TNL (TIR-NB-LRR) with an N-terminal Toll/interleukin-1 receptor domain (TIR) (6). Plant NLR proteins recognize the presence of pathogens either directly by binding to pathogen effectors, or indirectly by sensing effector-induced modification of other host proteins. The activation of ETI usually results in localized cell death at the infection site, which is referred to as a hypersensitive response (HR). The HR is commonly used as a read-out for the activation of NLR proteins in plants. The first NLR proteins, N and RPS2, were cloned in 1994 based on their ability to confer resistance to specific diseases in plants (7–9). However, the molecular mechanisms that control NLR activation and signaling remain poorly understood.

Here, we focus on the advances made in the last 2 years toward understanding how plant NLRs are activated and how signaling is initiated and transduced. We highlight the compartmentalization of plant NLRs, intra-/inter-molecular interactions before and after activation, and structural and genetic insights into NLR downstream signaling.

## Diverse Compartmentalization of Plant NLRs

**Figure 1:**
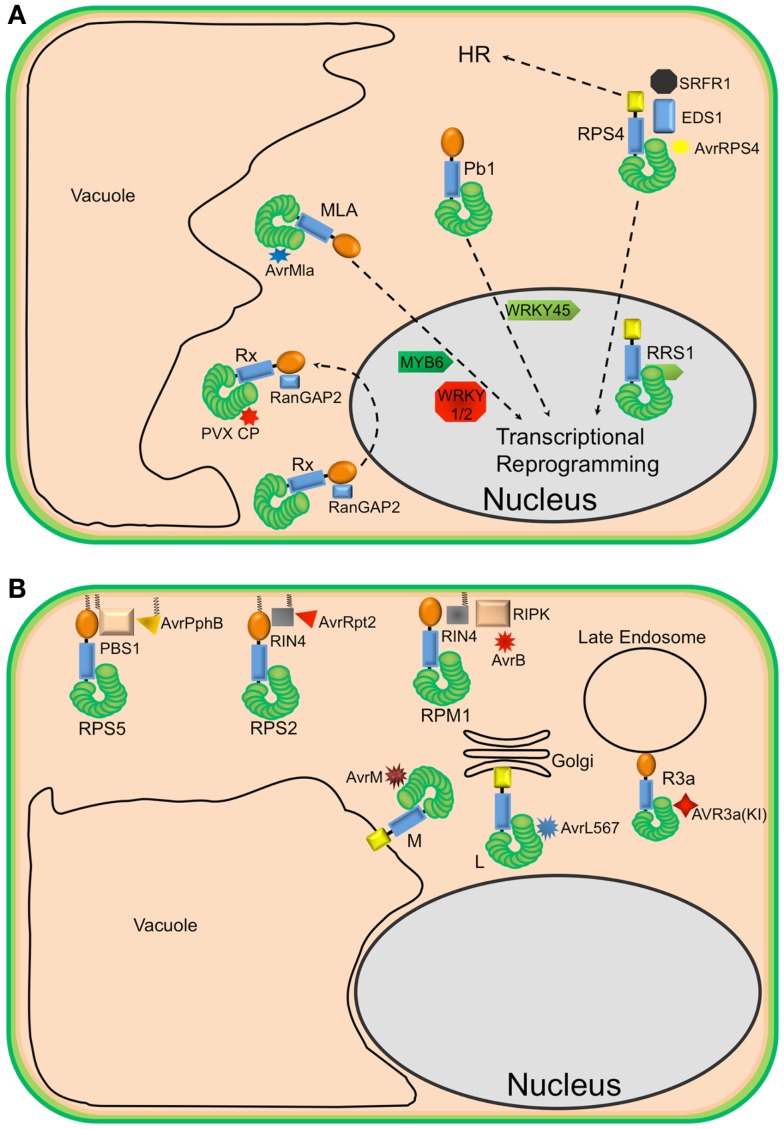
**Diverse localization of plant NLR proteins**. **(A)** Nuclear-localized plant NLRs. The barley MLA proteins reside in the cytoplasm but, in the presence of corresponding AvrMla effectors, translocate into the nucleus where they interact with both WRKY transcription repressors and MYB6, a transcriptional activator, to activate defense responses. Similarly, the rice CNL Pb1 also accumulates in the nucleus where it interacts with and stabilizes rice WRKY45 to activate defense responses. RPS4 also translocates into the nucleus, upon recognition of AvrRps4, to activate defense responses in conjunction with RRS1, an atypical TNL in *Arabidopsis* that contains a WRKY domain. At the same time, a subset of RPS4 complexes stays in the cytoplasm to activate HR. The potato CNL, Rx, interacts with the cytosolic Ran GTPase Activating Protein 2 (RanGAP2) and actively shuttles between the nucleus and the cytoplasm. However, the recognition of PVX CP and activation of signaling seem to occur in the cytoplasm. **(B)** Endomembrane associated plant NLRs and their corresponding “guardees” and pathogen effectors. RPS5 (an NLR), PBS1 (guardee), and AvrPphB (*P. syringae* effector) localize to the plasma membrane (PM). This is mediated by N-terminal acylation (myristoylation and/or palmitoylation). Similarly, RPS2 (an NLR) is PM-associated via a predicted N-terminal palmitoylation signal while RIN4 (guardee) localizes to the PM via a C-terminal prenylation or palmitoylation signal. RPM1 (NLR) also localizes to the PM, but lacks a predicted acylation signal. These three well-studied *Arabidopsis* NLR proteins are activated on the PM and initiate signaling on the PM. Relocalization following activation does not appear to occur. The flax rust resistance proteins L6 and M are respectively targeted to the Golgi apparatus and vacuolar membrane. Re-directing L6 to the vacuolar membrane, however, does not affect its function. The potato resistance protein, R3a, relocalizes from the cytoplasm to late endosomes in the presence of its corresponding effector AVR3a(KI), which also relocalizes to late endosomes in the presence of R3a.

The activation of NLR proteins is commonly associated with significant transcriptional reprograming. Consistent with this observation, several plant NLRs have been shown to accumulate in the nucleus upon effector-induced activation (10–13)(Figure 1A). For example, in the presence of the cognate powdery mildew effector AvrA10, the barley CNL, MLA10, translocates into the nucleus and interacts with both WRKY transcriptional repressors and MYB6, a transcriptional activator, to activate defense responses (10, 14). Similarly, nuclear accumulation of the *Arabidopsis* TNL, RPS4, is required for RPS4-mediated resistance in the presence of its cognate effector AvrRps4 (11, 15, 16). However, a number of recent studies have demonstrated that coordinated nucleo-cytoplasmic trafficking of plant NLRs is required for the full activation of defense responses, suggesting that a single NLR protein may activate distinct signaling pathways in the cytoplasm and nucleus. For example, the RPS4 protein of *Arabidopsis*, a TNL that mediates recognition of the effector protein AvrRps4 from *P. syringae*, appears to localize to both the nucleus and cytoplasm and activate different pathways in each. Forced nuclear accumulation of AvrRps4 is sufficient to activate RPS4-mediated bacterial growth inhibition, but blocks RPS4-mediated HR (16). On the other hand, sequestration of AvrRps4 in the cytosol using a nuclear export signal significantly impairs RPS4-mediated resistance but only moderately reduces RPS4-mediated HR. Therefore, nucleo-cytoplasmic partitioning of plant NLR proteins seems to be a regulatory mechanism for differential activation of downstream signaling. These studies also point out that activation of host cell death (HR) can be separated from activation of resistance.

Not all plant NLRs require nuclear localization for activation of resistance, and in fact, this may be the exception rather than the rule. The CNL protein, Rx, which mediates recognition of the Potato Virus X coat protein (CP), localizes to both the nucleus and cytosol (17, 18). Sequestration of Rx in the nucleus impairs its function, but forced cytosolic accumulation enhances Rx function (19). Moreover, Rx is not activated in the presence of forced nuclear PVX CP accumulation (20). Taken together, these results suggest that both pathogen recognition and resistance signaling by Rx need to take place in the cytoplasm. Thus, nuclear accumulation of Rx may represent a form of negative regulation. Alternatively, but not exclusively, Rx may have to traffic to the nucleus to form a functional complex and then back to the cytosol, where it surveys the presence of the cognate virus CP.

In contrast to Rx, RPS4, and MLA10, the CNL proteins RPS5 and RPM1 from *Arabidopsis* require PM localization to function (Figure 1B). This requirement likely reflects the localization of the pathogen effector proteins detected by each. RPS5 detects the *P. syringae* effector AvrPphB, which is a cysteine protease that targets the protein kinase PBS1.(21). AvrPphB autoprocesses upon entry into host cells, revealing an N-terminal motif that becomes myristoylated by host cell enzymes, which then targets AvrPphB to the PM (22). RPS5 is also acylated on its N-terminus and localizes to the PM (23). Mutation of the predicted acylation sites of RPS5 (Glycine 2 and Cysteine 4) disrupts RPS5-mediated HR and PM localization (23). Although RPM1 does not possess predicted acylation motifs at its N-terminus, it also localizes to the PM, where its corresponding effector AvrB and co-activators RIPK and RIN4 also localize (24–26). An auto-active RPM1 mutant, T166E, also localizes to the PM, indicating that RPM1 does not move following activation. Furthermore, sequestration of RPM1 on the PM does not affect RPM1-mediated resistance (25). Together, these observations indicate that activation of RPS5 and RPM1 and subsequent signaling occurs on the PM.

Plant NLR proteins have also been localized to other endomembrane locations. For example, the flax rust resistance proteins L6 and M localize to the Golgi apparatus and the tonoplast, respectively (27). Swapping the N-terminal sequences between L6 and M swapped their localization, indicating that the localization signals reside at the N-termini of these proteins, which are predicted to function as signal anchor sequences. Signal anchors are hydrophobic N-terminal sequences that direct nascent polypeptides to the endoplasmic reticulum, but unlike signal peptides, are not removed by a signal peptidase. Interestingly, changing the localization of L6 from the Golgi to the tonoplast did not affect its ability to detect its corresponding effector protein, nor activate resistance. Deletion of the signal anchor sequence, however, destabilized L6 protein accumulation, rendering it non-functional (27). A similar loss of protein stability was observed for RPS5 when its acylation motif was mutated (23), suggesting that at least a subset of NLR proteins require membrane localization for function and stability.

Plant NLRs can also move between the cytosol and endomembrane system. The potato resistance protein, R3a, relocates from the cytoplasm to endosomal compartments in the presence of the *Phytophthora infestans* effector AVR3a(KI) but not an unrecognized derivative AVR3a(EM) (28). Moreover, AVR3a(KI), but not AVR3a(EM), relocalizes to endosomes in the presence of R3a. Treatment with inhibitors of endocytic trafficking affects both the relocalization of R3a and its function. These observations suggest that the recognition of AVR3a(KI) by R3a and signal initiation occur in endocytic vesicles.

## Molecular Interactions during Plant NLR Activation and Signaling

The LRR domains are the most polymorphic part of plant NLR proteins, which likely reflects their role in effector recognition. Direct interaction between NLR proteins and pathogen effectors has been demonstrated for only a subset of plant NLRs, however. The best characterized of these is the flax L protein, in which allele specific interactions between L and its corresponding fungal effector AvrL567 have been demonstrated for the C-terminal LRR regions using yeast-two-hybrid analysis (29, 30). More recently, race-specific interactions between the *Arabidopsis* RPP1 LRR domain and the oomycete ATR1 effector have been demonstrated using co-immunoprecipitation analysis (31). However, race-specific physical interactions have also been shown between the coiled-coil (CC) domains of a rice NLR, Pik, and corresponding Avr-Pik effectors from the fungus *Magnaporthe oryzae* (32).

In addition to its role in effector recognition, the LRR domain also plays an important role in keeping NLR proteins in the “off” state. Studies of Bs2, RPS5, and Rx have demonstrated that the LRR domain physically associates with the NB-ARC domain (33–35). Furthermore, deletion of the LRR domain typically results in auto-activation (20, 35). A recent study on RPS5 established that only the first four LRRs are required to inhibit this auto-activation (23). Auto-activation has also been reported for the potato NLR Rx when its CC-NB-ARC region was co-expressed with RanGAP2 in tobacco plants (20). Auto-activation is also frequently observed when LRR domains are swapped between NLR proteins, suggesting that the LRR and NB-ARC domains co-evolve with each other (23, 36, 37). Consistent with this hypothesis, a highly acidic loop region in the Rx ARC2 domain has been shown to associate with basic patches in the N-terminal end of the Rx LRR domain (37).

The recently solved crystal structure of the mouse NLRC4 protein [NLR family, Caspase activation and recruitment domain (CARD) containing 4] provides additional insights into the physical interactions between the NB-ARC domain and the LRR domain that function to inhibit NLR auto-activation (38). NLRC4 displays an inverted “question-mark” structure, where the N-terminal region of the horse-shoe shaped LRR interacts with the NB subdomain of the NB-ARC. This interaction sterically restricts the accessibility of the side of the NB that is required for oligomerization. Deletion of the LRR domain, or point mutations in the NB/LRR interaction surface, result in constitutive activation of NLRC4 (38). The N-terminal region of the NLRC4 LRR domain also interacts with the ARC3 subdomain (also known as the helix domain 2), with this interaction playing an important role in the overall positioning of the LRR domain relative to the NB-ARC. Plant NLRs, however, do not contain an ARC3 subdomain (39), making it difficult to predict whether the LRRs of plant NLRs will be similarly positioned.

An open question in plant NLR studies is which domain(s) of plant NLRs is/are directly responsible for downstream signaling. In mammalian cells, the NLR activation usually results in the recruitment and activation of pro-caspase-1 through homotypic interaction with the N-terminal CARD (40). This leads to the formation of inflammasomes, which is linked to pyroptosis. By analogy, it is reasonable to assume that activation of plant NLRs exposes the N-terminal domain for downstream signaling. Indeed, overexpression of the N-terminal CC or TIR domains from two plant NLRs causes effector-independent HR, supporting a signaling role (41, 42). Crystal structures of the CC domain of the barley MLA10 CNL and the TIR domain of the flax L6 TNL indicate that homodimerization is necessary for downstream signaling activity (41, 42). In both studies, mutations at the dimer interface disrupted dimerization and signaling activity. However, mutations in the BB loop and αC helix of the L6 TIR domain did not affect homodimerization, but did disable downstream signaling, indicating the presence of discrete interfaces for self-association and engaging other unidentified signaling molecules (41). In addition, the presence of the L6 NB-ARC domain inhibited the dimerization of the L6 TIR and prevented signaling.

The above studies on L6 and MLA10 provide strong support for the CC and TIR domains functioning as the sole domains engaging downstream signaling components. However, conflicting data have been obtained from studies on the potato Rx and *Arabidopsis* RPS5 proteins. In the case of Rx, overexpression of the NB subdomain alone was found to be sufficient for inducing cell death, suggesting that this domain plays a roll in engaging downstream signaling components (43). For RPS5, overexpression of the CC or NB-ARC domains alone did not induce cell death, while overexpression of a CC-NB-ARC construct did, suggesting that the two domains function together to engage downstream components (35). It is not yet clear whether these conflicting data reflect fundamental differences between NLR proteins in terms of their signaling mechanisms, or are due to differences in how the experiments were conducted (e.g., different levels of overexpression, different epitope tags, etc.).

In addition to signaling, the N-terminal domains of plant NLRs may also function in effector recognition. For example, many effector targets, such as Pto, RIN4, PBS1, and NRIP, are found to interact with the N-terminal domains of their corresponding NLRs (44). Thus, the N-terminal domains of these NLRs may be responsible for directly monitoring effector-induced modifications of these target proteins or, alternatively, place their LRR domains in appropriate proximity for optimal surveillance. As mentioned above, race-specific interactions are reported to occur between the CC domains of the rice NLR, Pik, and corresponding Avr-Pik effectors (32). Similarly, L6 and L7 from the flax L locus recognize different effectors, but their amino acid sequences differ only in the N-terminal TIR domains (45).

Although direct association with pathogen effector proteins has been documented for some plant NLRs, many appear to detect pathogen effectors indirectly via sensing effector-induced modifications of other host proteins (46, 47). As mentioned above, the *Arabidopsis* CNL RPS5 detects the presence of the cysteine protease effector AvrPphB by monitoring the integrity of PBS1. In addition, insertion of seven amino acids at the AvrPphB cleavage site of PBS1 activates RPS5 as strongly as PBS1 cleavage, suggesting that RPS5 senses subtle conformational changes in PBS1 associated with its cleavage (48). Sensing of these structural changes by RPS5 is likely mediated by the LRR domain, as partial deletions as small as one LRR abolished activation by PBS1 cleavage, but did not abolish auto-activation by mutations in the NB-ARC domain (23). As a second example of indirect recognition, the *Arabidopsis* CNL, RPM1, detects modification of the *Arabidopsis* RIN4 protein induced by the *P. syringae* effector proteins AvrB and AvrRpm1. Current data indicate that AvrB physically associates with the *Arabidopsis* protein kinase RIPK, which then phosphorylates RIN4 (26). The phosphorylation of specific RIN4 residues then leads to the activation of RPM1 (24, 26). AvrRpm1 appears to induce modification of other RIN4 residues, but the specific residue(s) modified, and whether it is by phosphorylation, is not yet clear. A third example of indirect recognition of pathogen effectors is recognition of the *P. syringae* effector AvrRpt2 (a cysteine protease) by *Arabidopsis* RPS2. In this example, RPS2 is activated by the degradation of RIN4 following cleavage by AvrRpt2 (49). Thus RIN4 is required to keep RPS2 in an off state, and *rin4* null mutations are lethal in *Arabidopsis* if *RPS2* is functional.

## Nucleotide Binding and Oligomerization

The NB-ARC domain is conserved among plant and animal NLRs, and in the animal literature is often referred to as the nucleotide binding and oligomerization domain (NOD). NB-ARC domains form a deep nucleotide-binding pocket. In the “off” state, the NB-ARC domain adopts a “closed” structure where ADP is preferentially bound and coordinates intramolecular interactions to stabilize this structure (38, 50, 51). Activation is thought to require release of the ADP to be replaced by ATP and adoption of an “open” structure. This structural change is then thought to promote homo-oligomerization via the NB-ARC domain, which in turn enables the N-terminal domains to engage in downstream signaling. Thus, the NB-ARC domain is thought to function as a molecular switch that determines the “on” and “off” state of NLR signaling with ADP bound form for “off” and ATP bound form for “on.” Due to difficulties in purifying soluble plant NLR proteins, however, this long-standing model was not tested until recently. Biochemical studies using the CC-NB-ARC region of tomato I-2 and Mi-1 demonstrated that ATP is bound by these proteins (52). This binding activity depends on a functional P-loop (Phosphate-binding loop), also known as the Walker A motif, which is a glycine-rich flexible loop containing a highly conserved lysine residue that interacts with the phosphates of the nucleotide and with a magnesium cation that coordinates the β- and γ-phosphates (53). In addition, these proteins display ATPase activity. A follow-up study reported that mutations within the NB-ARC domain that cause an auto-activation phenotype impair the ATPase activity of I-2, supporting the model that the ATP bound form represents the “on” state (54). The first direct experimental evidence that a full-length plant NLR protein preferentially binds ADP in its resting state was reported in 2011. In that study, the barley CNL, MLA27, co-purified specifically with ADP but not ATP (42). Also in 2011, the flax rust resistance protein M was found to co-purify with ADP, while an auto-active mutant form (D555V in the conserved MHD motif) co-purified with more ATP than ADP (55), supporting the model that nucleotide exchange is required for switching from the off state to the “on” state.

Apaf-1 and CED-4 are known to form oligomers through NB-ARC-mediated interaction (51, 56). The crystal structure of the mouse NLRC4 protein mentioned above revealed that ADP coordinates interactions between the central NB subdomain and the ARC2 subdomain (also called the winged-helix domain) to stabilize a closed conformation. A second interaction surface between the ARC3 subdomain and the NB masks an α-helix of the NB subdomain that participates in oligomerization. This α-helix is part of a conserved structure within the STAND family of ATPases (38). Specific point mutations within these interaction surfaces also result in an auto-active phenotype. A structure for NLRC4 in an active confirmation is not yet available, but it is predicted that ligand binding leads to the disengagement of the ARC2, ARC3, and LRR domains from the NB simultaneously, allowing the oligomerization of NLRC4 mediated by the NB subdomain.

There are several reports that indicate plant NLR proteins can also self-associate. For example, tomato Prf forms a dimer, which then incorporates into a complex containing two accessory molecules of the Pto protein kinase (57, 58). Similarly, co-immunoprecipitation assays have demonstrated that *Arabidopsis* RPS5 self-associates prior to activation (35). Oligomer formation has also been reported for the tobacco TNL protein, N, but only its N-terminal TIR domain has been associated with oligomerization (59). The crystal structures of the MLA10 CC and L6 TIR domains revealed that both form homodimers (41, 42), and the MLA1 protein (allelic to MLA10) was observed to self-associate *in planta* (42). Whether the NB-ARC domain also plays a role in plant NLR oligomerization remains unclear, however. So far, only the RPS5 NB-ARC domain is known to self-associate, and this was shown under conditions of transient overexpression (35). In contrast, the L6 NB-ARC domain was shown to inhibit the homodimerization of its TIR domain and the activation of defense responses (41). ATP binding seems to be a must, however, for oligomerization because P-loop mutations disrupted the formation of N oligomers (59).

In addition to self-association, different NLRs may interact with each other to form heterodimers or hetero-oligomers. Interaction between NLRs has been reported between mouse NLRC4 and NAIP2, and between NLRC4 and NAIP5 (60, 61). NLRC4-containing oligomers assemble in response to two distinct PAMPs, flagellin and PrgJ, a component of type III secretion systems (40, 62). However, NLRC4 does not directly interact with flagellin or PrgJ. Instead, these PAMPs bind to NAIP2 and NAIP5, respectively, which then bind to NLRC4 (60, 61), triggering formation of a functional NLRC4 inflammasome. Like NAIP2 and NAIP5, some plant NLRs appear to require a second NLR for signaling. The *Arabidopsis* NLR RPS4 requires a second NLR, RRS1, to recognize the AvrRps4 effector protein from *P. syringae* (63, 64). Interestingly, RRS1 is encoded by a gene immediately adjacent to *RPS4*, in a head-to-head orientation (63, 64). This pair of NLRs is also involved in recognition of the PopP2 effector protein from the bacterial pathogen *Ralstonia solanacearum* and an unidentified effector(s) from the fungus *Colletotrichum higginsianum*. It is not yet known whether RRS1 and RPS4 physically associate, but both at least partially localize to the nucleus in the presence of effectors (11, 65).

A second example of a “helper” NLR is the *Arabidopsis* ADR1 (Activated Disease Resistance 1) family, which contributes to defense responses activated by *Arabidopsis* RPS2 (a CNL), RPP2, and RPP4 (TNLs) (66). There are three copies of ADR1 in the *Arabidopsis* genome and all three must be knocked out to affect RPS2 signaling. Interestingly, ADR1 does not rely on an intact P-loop motif for this function, suggesting that ATP binding is not required for signaling. ADR1 family members are also required for basal resistance and PTI, suggesting that this family of CNLs may function more generally in regulating defense responses, rather than functioning specifically in effector detection. Consistent with this, attempts at showing direct physical interactions between ADR1 and other NLRs have not been successful (66). In addition, mutations in ADR1 family members suppress runaway cell death triggered by loss of the *LSD1* gene, and autoactivating mutations in ADR1-L2 cause large increases in the defense hormone salicylic acid (67). Together, these data suggest that ADR1 may be part of an amplification loop that leads to elevated levels of salicylic acid during defense responses.

An ADR1 homolog has also been described in tobacco, and has been named NRG1. The NRG1 protein is required for resistance mediated by the TNL protein N, which mediates recognition of tobacco mosaic virus (TMV) (68). Consistent with their proposed role in signaling, transient expression of the CC domains of NRG1 and ADR1 induces HR in tobacco plants (69). NRG1 and ADR1 belong to an ancient clade of CNLs that is unusually conserved relative to other plant NLR proteins. Phylogenetic analyses have revealed the correlated absence of both NRG1 homologs and TIR-NB-LRR-encoding genes from the dicot *Aquilegia caerulea* and the dicotyledonous order Lamiales, as well as from the grass family (Poaceae), suggesting that the TNL family may be dependent on ADR1 family members for activating resistance (69). Since grasses contain numerous CNL family members, this functional requirement appears not to be true for CNLs in general. Indeed, resistance mediated by the *Arabidopsis* RPM1 protein is not affected by loss of ADR1 function (66).

## Downstream Signal Transduction and Defense Activation

It has been almost 20 years since the cloning of the first plant NLR gene. During this period, major advances have been made in our understanding of NLR structure, activation, and localization. However, little is known about the signal transduction steps following plant NLR activation. Forward genetic screens have been mostly unsuccessful at identifying downstream components, likely due to redundancy of signaling pathways. One exception was the identification of EDS1 (enhanced disease susceptibility 1) in *Arabidopsis*, which is required for resistance mediated by TNLs but not CNLs (70, 71). EDS1 has recently been shown to form protein complexes with the *Arabidopsis* TNLs RPS4, RPS6, and SNC1 (72). These complexes also contain an unrelated protein named SRFR1, which was identified in a screen for mutations that restored resistance to *rps4* mutant *Arabidopsis* (73, 74). Furthermore, the bacterial effectors recognized by RPS4 and RPS6 (AvrRps4 and HopA1) bind to EDS1 and disrupt EDS1-SRFR1 interactions (72). This study suggests that EDS1 may be a “guardee” of RPS4 and RPS6 (and possibly other TNLs) and that these TNLs are activated by effector mediated disruption of the EDS1-SRFR1 complex. More recent work, however, found that the C-terminal half of AvrRps4, which is necessary and sufficient for activating RPS4, does not interact with EDS1 in co-immunoprecipitation or yeast two-hybrid assays (75). This finding suggests that physical association between AvrRps4 and EDS1 is not required for activation of RPS4, thus the molecular mechanism underlying AvrRps4 recognition remains unclear.

Regardless of whether EDS1 is a true target of AvrRps4, it is clear that EDS1 and SRFR1 represent a signaling complex that is employed by multiple TNLs. SRFR1 contains a tetratricopeptide repeat domain and displays similarities to transcriptional repressors in *Saccharomyces cerevisiae* and *Caenorhabditis elegans* (74). Consistent with SRFR1 possibly functioning as a transcriptional repressor, two independent studies reported that loss of SRFR1 function activates the expression of SNC1, an *Arabidopsis* TNL, resulting in constitutive defense responses (76, 77). Furthermore, bifluorescence complementation analyses showed that SRFR1 interacts with RPS4 and SNC1 in the nucleus (72), suggesting that TNLs may directly regulate SRFR1 activity.

Other transcriptional regulators have also been shown to directly interact with TNLs. For example, Topless-related 1 (TPR1) interacts with SNC1, a TNL protein, and knocking out TPR1 compromises immunity mediated by SNC1 (78). Significantly, TPR1 represses the expression of two well-known negative regulators of immunity, Defense no Death 1 (DND1) and Defense no Death 2 (DND2). Therefore, the SNC1-mediated immune responses are activated by TPR1 through its repression of negative regulators. SPL6, a squamosa promoter binding protein (SBP)-domain transcription factor interacts with the N protein of tobacco within distinct nuclear compartments (79). The *Arabidopsis* ortholog of SPL6 is required for the RPS4-mediated resistance, indicating that this transcription factor plays a conserved role in activating TNL-mediated defenses. Also, as described above, the CNL protein MLA 10 translocates into the nucleus upon activation and interacts with both WRKY transcriptional repressors and MYB6, a transcriptional activator, to activate defense responses (10, 14). Most recently, the rice CNL Pb1, which confers resistance to rice blast (*Magnaporthe oryzae*), was shown to interact with the WRKY45 transcription factor in the nucleus (80). This interaction is mediated by the CC domain of Pb1, and mutations in the CC domain that disrupt the interaction compromise Pb1-mediated resistance. Thus both CNLs and TNLs have the capacity to impact gene expression by direct interaction with transcriptional repressors and activators, making these NLR signal transduction pathways quite short.

It is unlikely, however, that all NLR proteins regulate gene expression by direct interaction with transcription factors. As described above, the CNL proteins RPS2, RPS5, and RPM1 are localized to the PM. Activation of defenses by PM-localized NLRs appears to require an influx of extracellular Ca^2+^, as cell death induced by RPS2 and RPM1 can be eliminated by the calcium channel blocker LaCl_3_ (81). Recent reverse genetic studies indicate that RPS2- and RPM1-mediated resistance is at least partially dependent on calcium dependent protein kinases (CPKs) (82, 83), with different CPKs being involved in different aspects of resistance (83). Specifically CPK1 and CPK2 contribute to HR development, while CPK4/5/6/11 all contribute to transcriptional reprograming by phosphorylating the transcription factors WRKY8/28/48. Additionally, CPK1/2/4/11 also contribute to production of reactive oxygen species via phosphorylation of PM-associated NADPH oxidases. Based on these observations, the authors proposed a model in which NLR activation triggers a sustained influx of calcium, which then triggers multiple CPK signaling pathways that lead to ROS production, defense gene activation, and cell death. In addition to cytoplasmic calcium signaling, RPS2 and RPM1 activation has been shown to elicit specific Ca^2+^ signatures inside chloroplasts (84). These calcium transients are dependent on a chloroplast-localized protein named CAS for calcium-sensing receptor. Mutations in the *CAS* gene compromise both PTI and HR development during ETI. This study thus provides a possible link between NLR activation and chloroplast functions such as the production of the defense-related hormones jasmonic acid and salicylic acid.

Although it is clear that different plant NLRs employ different signaling pathways, these signaling pathways appear to be broadly conserved across plant species, as evidenced by functional transfer of NLRs between species. For example, the *RRS1-RPS4* gene pair described above has been functionally transferred from *Arabidopsis* to five different plant species from three different families (*Brassica rapa* and *Brassica napus* (Brassicaceae); *Nicotiana benthamiana* and *Solanum lycopersicum* (tomato) (Solanaceae), and *Cucumis sativus* (cucumber, Cucurbitaceae) (85). In addition, cell death can be activated in *N. benthamiana* and/or *N. tabacum* (tobacco) by transient expression of several different TNL and CNL proteins from diverse plant species, including *Arabidopsis*, flax, and barley (35, 86–88). Particularly noteworthy is the recent demonstration that the MLA1 protein from barely can function in transgenic *Arabidopsis* to confer resistance against the barley powdery mildew fungus, *Blumeria graminis* f. sp. *hordei* (89). Interestingly, this resistance remains effective in *Arabidopsis* mutants defective in ethylene, jasmonic acid, and salicylic acid signaling, indicating the presence of a hormone independent NLR-mediated defense mechanism that is conserved between barley (a monocot and member of the grass family) and *Arabidopsis* (a dicot and member of the mustard family).

The HR is usually associated with NLR-activated immunity in plants. However, the HR can be genetically uncoupled from restriction of pathogen growth, at least in the case of resistance to *P. syringae* (16, 90). In addition, it remains unclear how cell death is executed, or indeed, whether different classes of NLRs share the same cell death pathway. For the PM-localized NLRs, RPM1, and RPS2, cell death is preceded by fusion of the vacuolar membrane with the PM, resulting in release of vacuolar proteins to the apoplast (extracellular space) (91). The resulting extracellular fluid possesses both antibacterial activity and cell death-inducing activity. This membrane fusion process depends on the activity of the proteasome subunit PBA1, suggesting that there may be an “HR inhibitor” protein that must be degraded to enable HR activation.

Plants lack canonical caspase proteases that are associated with apoptosis in mammalian cells. However, they do contain proteins with weak structural similarities to caspases called metacaspases that have recently been implicated in regulating HR cell death (90). Knockout of the *Arabidopsis* metacaspase AtMC1 reduces, but does not eliminate, RPM1-mediated HR, but has no effect on RPM1-mediated growth restriction of *P. syringae*. Conversely, knockout of a second *Arabidopsis* metacaspase AtMC2, enhances RPM1-mediated HR, but again has no effect on restricting bacterial growth. These observations suggest that metacaspases play an accessory role in regulating HR, but are not a central trigger.

A second type of protease associated with HR regulation in plants has recently been identified and named phytaspase (90, 92). Phytaspases are structurally unrelated to animal caspases, but like caspases, catalyze cleavage following aspartate residues. RNAi-mediated silencing of phytaspase in tobacco reduced N-gene mediated HR triggered by TMV infection and reduced resistance to TMV, indicating that phytaspases may play a central role in resistance mediated by N, a TNL family member (90, 92). Interestingly, tobacco phytaspase is constitutively expressed and secreted to the extracellular space, but during the HR, partially relocalizes to the cytoplasm (90, 92), raising the possibility that it is actively transported back into the cell during the HR, where it must cleave specific substrates to activate cell death. Although phytaspase has also been purified from rice, there are not yet any reports on whether it is required for NLR-mediated resistance in other plant species.

Several recent studies indicate that secretion may play an important role in NLR-mediated defense. For example, *Arabidopsis* AtMIN7, an ADP ribosylation factor-guanine nucleotide exchange factor (ARF-GEF) protein, has recently been shown to be required for RPS2- and RPS5-mediated resistance, but not for HR cell death (93). ARF-GEF proteins regulate the activity of small GTPases involved in endomembrane trafficking. AtMIN7 is a target of the *P. syringae* effector, HopM1, which promotes proteasome-dependent degradation of AtMIN7 (94). Activation of RPS2 and RPS5 somehow prevents HopM1-mediated degradation of AtMIN7 (93). Consistent with AtMIN7 playing a role in endomembrane trafficking, confocal microscopy showed that MIN7 and HopM1 localize to the *trans*-Golgi network/early endosomes. Further evidence that endomembrane trafficking/secretion plays a role in RPS2-mediated resistance comes from quantitative proteomic analysis of PMs following RPS2 activation (95). In this study, a transgenic *Arabidopsis* line expressing a dexamethasone-inducible AvrRpt2 gene was used to activate RPS2. Comparison of activated to unactivated samples uncovered 235 proteins that were significantly up-regulated. This set of up-regulated proteins was highly enriched in proteins involved in endocytosis and exocytosis, including Syntaxin of plants 122 (SYP122) and *N*-ethylmaleimide-sensitive factor vesicle fusing ATPase, and soluble *N*-ethylmaleimide-sensitive factor adaptor protein 33 (SNAP33). RPS2 has also been shown to upregulate production of miR393b, a microRNA that targets at least three different genes likely involved in endomembrane trafficking (MEMB12, a golgi-localized SNARE protein; VPS54, homologous to a yeast protein involved in retrograde transport from late endosomes to the Golgi, and EXO70H3, a subunit of the exocyst complex thought to be required for exocytosis (96). Knockout of MEMB12 enhances secretion of the defense protein PR-1 that is induced by RPS2 activation. Thus MEMB12 appears to function as negative regulator of exocytosis, with RPS2 inducing production of a miRNA that inhibits translation of the MEMB12 protein. Lower MEMB12 protein levels then enable an increase in defense protein secretion. Consistent with this model, the MEMB12 knockout line displays enhanced basal resistance in the absence of RPS2 activation (96). The endomembrane trafficking system is thus emerging as important arm of the NLR-mediated defense system that is also targeted by pathogen effectors.

## Prospective

As should be apparent from the discussion above, plant NLRs have evolved diverse mechanisms for recognizing pathogens and diverse mechanisms for activating resistance. However, a feature that is likely shared among all “sensor” NLRs in plants (as opposed to “helper” NLRs) is the dual role of the LRR domain in keeping the NLR in the “off state” in the absence of pathogen, and promoting the switch to the “on state” in the presence of pathogen (via binding to effectors or effector-modified host proteins). We have very little insight, however, into how the LRR domain accomplishes either of these roles. The recent structure of mouse NLRC4 indicates that in animal NLRs, the LRR folds back across NB-ARC domain with the N-terminal portion of the LRR making multiple contacts with the NB and ARC3 domains, effectively placing a lid over the ADP/ATP binding pocket. The absence of the ARC3 domain in plant NLRs makes it a certainty that the contacts between the LRR and NB-ARC will differ from NLRC4, but based on the locations of autoactivating mutations and on deletion analyses, the general structure is likely to be similar, with just the N-terminal portion of the LRR (approximately four repeats) required to form the lid (23). The C-terminal portion of the LRR appears to be where specificity for effector recognition generally lies, but how effector binding alters NB-ARC:LRR interaction is unknown. What remains a holy grail for the field, in both plants and animals, is obtaining the structure of an NLR complexed with its activating protein. The insolubility of NLRs when overexpressed in bacteria or insect cells has been a major barrier to progress on this front. Surmounting this barrier for plant NLRs may require purification from plant systems in which the necessary chaperones should be present.

A second holy grail is identifying the immediate downstream interacting proteins for PM associated CNLs. Although several transcription factors have now been identified that interact with nuclear-localized TNLs and CNLs, we still lack good candidates for downstream signaling proteins for NLRs that signal from the PM such as RPM1, RPS2, and RPS5. Proteomic approaches hold some promise for shedding light on this unknown (95, 97), but face the additional challenge of rapid turnover of NLR proteins following activation. The finding that extracellular calcium influx is required for RPM1- and RPS2-mediated HR suggests that there may be a fairly direct link between NLR activation and calcium channels (81), which merits further exploration.

A third holy grail is a better understanding of how cell death is executed during NLR-mediated HR. Although cell death is apparently not required for resistance, at least to *P. syringae*, the HR is still a hallmark of NLR activation. One study has implicated fusion of the vacuolar and PM as the primary event leading to cell death (91). If this is true, the question becomes how NLR activation triggers such membrane fusion events. More generally, accumulating data have implicated endomembrane trafficking as playing a central role in NLR-mediated resistance, presumably to increase secretion of antimicrobial compounds. How does NLR activation regulate this process?

In summary, although much has been learned in the nearly 20 years since the first NLR was identified, major questions remain. Providing answers to these questions will require both creativity and improvements in technology, but will no doubt come.

## Conflict of Interest Statement

The authors declare that the research was conducted in the absence of any commercial or financial relationships that could be construed as a potential conflict of interest.
